# Evaluation of serum zinc levels in patients with recurrent aphthous stomatitis (RAS)

**DOI:** 10.1186/s12903-017-0450-x

**Published:** 2017-12-20

**Authors:** Zuzanna Ślebioda, Ewa Krawiecka, Elżbieta Szponar, Barbara Dorocka-Bobkowska

**Affiliations:** Department of Gerodontology and Oral Pathology, University of Medical Sciences, ul. Bukowska 70, 60-812 Poznań, Poland

**Keywords:** Recurrent aphthous stomatitis, Zinc, Oral mucosa, Oral diseases

## Abstract

**Background:**

Recurrent aphthous stomatitis (RAS) is an ulcerative disease of the oral mucosa without a clearly defined etiology. The aim of the study was to evaluate the serum zinc levels in patients with RAS in comparison to healthy controls and to validate the association between zinc levels and the course of RAS.

**Methods:**

Seventy-five patients with RAS and 72 controls underwent full dental examination. Serum zinc levels were determined by flame atomic absorption spectroscopy (F AAS). The results were statistically analyzed with Kruskal-Wallis, Mann-Whitney, chi-square tests and the test of difference between the two rates of structure with *p* < 0.05 as a significance level (Statistica 10, StatSoft®).

**Results:**

No statistically significant differences were detected in serum zinc levels between RAS patients and healthy controls. The mean serum zinc concentration was found to be 84.2 μg/dL in RAS group and 83.9 μd/dL in controls, within the accepted norms. Zinc deficiency was observed in 10.7% patients from the RAS group and in 6.9% controls. No significant differences in serum zinc levels were found between patients when the course of the disease was considered.

**Conclusions:**

Serum zinc concentrations did not differ significantly in RAS patients and in healthy controls and it did not influence the course of the disease. Therefore, zinc does not appear to be an important modifying factor in the development of RAS.

## Background

Recurrent aphthous stomatitis (RAS) is a chronic, ulcerative condition of the oral mucosa without a fully recognized etiology [1, 2]. The current research suggests that genetically conditioned abnormal immune response triggered by various local and systemic factors, plays an essential role in the disease [1, 3]. An abnormally initiated cascade of proinflammatory cytokines acting against the host mucosa leads to the formation of massive leukocytic infiltration and local tissue damage [1]. Disruption of the humoral and cellular immune response in patients with RAS results in an elevated concentration of a complement of species: increased number of NK (natural killer) cells and B lymphocytes, disrupted CD4/CD8 (cluster of differentiation 4/8) ratio, increased number of CD25 (cluster of differentiation 25) and TCR (T cell receptor) ɣδ cells in peripheral blood and hyper-reactivity of neutrophils [4–6].

RAS is characterized by the presence of painful, oval erosions or ulcers generally localized on the unattached oral mucosa of the lips, cheeks and tongue, which are surrounded by an erythematous halo. Lesions reappear frequently and to date now there has been no effective causative treatment available [1–3]. According to Stanley, the three main types of the disease can be described: major (MaRAS), minor (MiRAS) and herpetiform (HeRAS). The classification criteria include the size and the depth of the lesion, the number of lesions in one episode, their location and duration [1]. Most commonly observed type is minor, where the diameter of lesion varies between 5 and 10 mm. The number of eruptions per one flare-up in this type does not normally exceed 10, while the healing lasts for 10 to 14 days. This form affects from 75 to 90% of all patients with RAS. Major type is found in 10 to 15% of RAS subjects and usually develops as a single ulcer with a diameter over 1 cm, located on keratinized or non-keratinized oral mucosa. The healing phase lasts longer than in case of MiRAS and may take up to 1 month. This form is considered as the most severe one. The least common type is herpetiform RAS, where the crucial finding is the presence of multiple, small, short-lasting erosions that spread throughout the oral cavity, healing within 14 days without leaving a scar [1, 2]. The presence of recurrent oral aphthae accompanied by genital ulcers and uveitis is a characteristic of a systemic condition called Behçet’s disease, which may also involve some other visceral organs [7, 8].

Depending on the diagnostic criteria, the condition affects between 10 and 20% of the general population. The peak period of the RAS occurrence is the second life decade. A higher incidence among females, non-smokers, white races and people with a high socio-economic status has also been reported in some studies [1, 2].

**Fig. 1:**
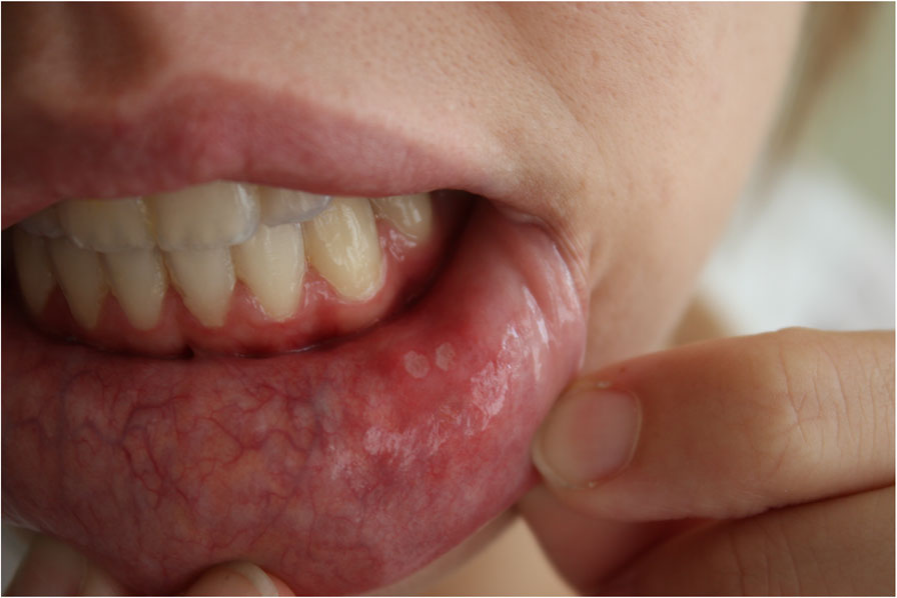
Minor aphthae on the lower lip

Minor aphthae on the lower lip are presented on Fig. 1.

Zinc is an essential, biologically active, trace metal in the human body. It acts as co-enzymatic and activator of almost 100 human enzymes. It participates in the metabolism of lipids, proteins and carbohydrates, regulates RNA and DNA biosynthesis, modifies the growth and development of epithelium and helps to maintain the correct serum vitamin A concentration [9–11]. Zinc levels have been shown to affect the immune response; for example in Zn deficiency, interleukin 2 (IL-2) secretion is limited and the number of T1- helper lymphocytes (Th1) is reduced. Zinc exhibits anti-oxidative properties by inhibiting the oxidation of unsaturated fatty acids. Zinc deficiency is characterized by a broad spectrum of clinical symptoms that include: impaired growth and maturation, loss of appetite and insufficient body weight, pathologic skin lesions (eczema, stretch striae), impaired healing, vision disturbances, signs of accelerated aging, psychiatric and neurosensoric disturbances, reduced resistance to infections and elevated risk of diabetes due to increased glucose tolerance [9–12]. Zinc deficiency has been reported in patients with various systemic conditions such as: psoriasis, acne vulgaris, pernicious anemia, tuberculosis and cirrhosis [12–16].

A correlation of zinc levels in human body with some oral mucosa diseases has been already suggested, especially those with a potential autoimmune background, such as lichen planus and geographic tongue [16–21]. Although the etiology of RAS remains still not fully recognized, the presence of antibodies for different antigens of the epithelium in patients with aphthae also suggests an autoimmune character of the disease [3]. The role of several immunomodulators in the development of the condition has been recently considered. Primary immune abnormalities and immune system disruption observed in RAS patients may partially result from nutrient deficiencies that include iron, folic acid, and vitamin B group [1, 2, 11, 14]. The potential role of zinc deficiency as the modifier of the RAS course may result from the ability of zinc to stimulate the production of IL-1, IL-6 and TNF-α in peripheral blood mononuclear cells and separated monocytes. It was observed that the cytokines production becomes disrupted in zinc deficient subjects- low zinc serum levels correlate with a reduced production of Th1-type cytokines [9]. Meanwhile, the local tissue damage at the initial stage of aphtha formation occurs in the response to an abnormally stimulated cascade of cytokines [3, 4]. Several authors also suggest that Th1-type immunologic response plays an essential role in the etiopathogenesis of RAS [3]. Therefore recently, due to its proven immunoregulating and regenerating properties, zinc has raised a considerable interest as the potential trigger of the RAS development and modifier of its course. Deviations in serum zinc levels could on one hand influence the risk of the disease, but on the other hand it could also affect the course of RAS by promoting more severe forms like MaRAS in zinc deficient subjects.

The aim of the present study was to compare serum zinc levels in patients with RAS and in a group of healthy controls and to evaluate its effects on the disease severity.

## Methods

This is a case-control study conducted in Poznan University of Medical Sciences (PUMS) in Poland between September 2013 and April 2015. Seventy five patients with RAS from the Great Poland region, including 46 females and 29 males with a mean age of 35.08 ± 16.9 years were enrolled in the study. All had at least a 1-year history of the disease and a regular mode of recurrences, defined as at least two episodes per year. The control group consisted of 72 participants with no evidence and/or clinical history of RAS, 53 females and 19 males with a mean age 32.2 ± 14.3 years, recruited from the patients, students and stuff members of PUMS. Demographic characteristic of the study and control group is presented in Table 1.

**Table 1:** Characteristic of the RAS and control group

	Total	Age	Mean ± SD	Females	♀Age	Mean ± SD	Males	♂Age	Mean ± SD
RAS	75	8–82	35 ± 17	46	9–82	34 ± 17	29	8–68	37 ± 17
Control	72	1–79	31 ± 15	53	19–79	33 ± 14	19	1–68	31 ± 17
P			0.4215			0.9805			0.2853

The exclusion criteria included the presence of an ulcerative oral mucosa disease other than RAS and current immunosuppressive or immunomodulatory treatment. After recording a detailed medical history of all the participants, a full extra- and intraoral examination was performed by a qualified dental specialist (ZŚ). RAS was confirmed in all of the subjects from the study group during the visit. Depending on the type of aphthae, patients from the study group were divided into MiRAS, MaRAS and HeRAS subgroups. Diagnostic criteria included the size of the lesion, number of eruptions in a single flare-up and healing time. Additionally, based on the mode of recurrences, subjects were assigned to a mild, moderate or severe RAS course subgroup. The latter classification, based on the patients’ self-report, was proposed by Bagan et al.; type 1 disease is characterized by the intervals between the flare-ups of over 3 months, while in the type 2 disease the flare-ups occur at one to 3 month intervals. In type 3 aphthous lesions are present almost continuously [22]. Depending on the number of lesions per episode, the RAS patients were classified as group A (1–3 lesions per episode) or group B (over 3 lesions per episode). Blood samples were collected to determine the serum zinc concentration in all participants. The zinc concentration was measured by flame atomic absorption spectroscopy (F AAS; Jena AG; ZEEnit700E) using an analytical method described by Arora et al. [12], Khademi and Shaikhiany [19], Orbak et al. [23] and Özler [24]. The results were statistically analyzed with non-parametric tests, as the data were not normally distributed according to Shapiro-Wilk normality test. Kruskal-Wallis test was utilized to compare mean serum zinc levels in Mi-, Ma- and HeRAS subgroups as in mild, moderate and severe RAS subgroups. We used Mann-Whitney test to compare the mean serum zinc levels in A and B subgroups. Chi-square test was used to assess the independence of variables between the study subgroups and the serum zinc level described as low, normal or high. The test of difference between the two rates of structure was utilized to compare the frequency of zinc deficiency between the study subgroups. In all tests *p* < 0.05 was considered as a significance level (Statistica 10, StatSoft®).

## Results

The mean serum zinc concentration was found to be 84.2 ± 13.49 μg/dL in the RAS group and 83.9 ± 10 μg/dL in the control group. The difference assessed by Mann-Whitney test was not statistically significant (*p* = 0.7154). Zinc deficiency defined as a serum Zn concentration < 70 μg/dl was observed in 8 (10.7%) patients with RAS and in 5 (6.9%) of the control group (the difference was statistically insignificant; *p* = 0.4259). Low serum zinc concentrations defined as Zn level between 70 and 80 μd/dl was found in 18 (26.7%) of RAS patients and in 20 (25.0%) of the control group which was statistically insignificant difference (*p* = 0.8246). There was also no significant difference between RAS and controls when a combined study group (“deficiency” + “low zinc” subgroups) was considered (26 patients; 37.3% in RAS group vs. 25 patients; 31.9% in control group; *p* = 0.4669). However, generally lower zinc concentrations were observed in the RAS group compared to the control group. Zinc concentrations above the upper norm were observed in 2 RAS patients (1.4%) and in none of the control group. No differentiation between the study groups was apparent in terms of zinc serum concentrations as determined by the chi-square test analysis (*p* = 0.4061).

The most commonly observed type of aphthae in the study group was MiRAS (56 patients, 74.7%). MaRAS was found in 14 (18.7%) and HeRAS in 5 (6.7%) subjects. The mean serum zinc concentrations in MiRAS, MaRAS and HeRAS subgroups were 83.5 ± 10.2 μg/dL, 80 ± 10.15 μg/dL and 103.8 ± 32.3 μg/d, respectively, but the differences were considered statistically insignificant (*p* = 0.2855). Low zinc levels or zinc deficiency was revealed in 21 (37.5%) MiRAS patients, 6 (42.9%) MaRAS and in 1 (20%) HeRAS subject. The differences in frequency of low Zn and Zn deficiency between the most numerous MiRAS subgroup and the subgroups with other types of aphthae was considered statistically insignificant (*p* = 0.8730).

Based on the frequency of episodes the course of RAS was classified as mild (episodes with more than 3-month intervals), observed in 27 (36%) patients, moderate (episodes with 1–3 month intervals), found in 33 (44%) subjects and severe (lesions present almost constantly), revealed in 15 (20%) patients from the study group. The mean serum zinc concentrations in these subgroups were: 87.6 ± 18.8 μg/dL, 82.1 ± 7.5 μg/dL and 82.8 ± 12.1 μg/dL, respectively. The differences between these levels were not considered significant (*p* = 0.5032). There was no association between the course of RAS and serum zinc concentration (*p* = 0.3912). Low zinc levels or zinc deficiency occurred in 9 (33.3%) patients with mild-type RAS, in 14 (42.4%) moderate-type RAS patients and in 5 (33.3%) subjects with severe type of RAS. The differences were also considered to be statistically insignificant.

**Fig. 2:**
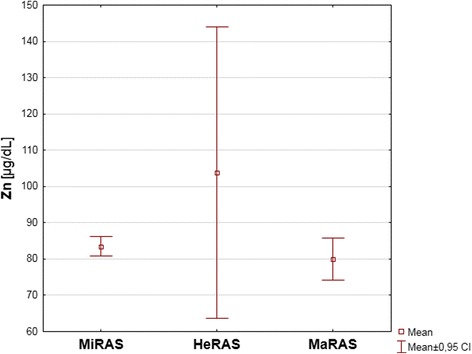
Mean serum zinc levels in patients with Mi-, Ma- and HeRAS

Fifty-nine RAS patients (78.7%) were assigned to the A subgroup, where 1 to 3 lesions appeared during one episode. The remaining 16 subjects (21.3%) were classified as B subgroup with over 3 lesions per episode. The mean serum zinc concentrations in these subgroups were: 83.1 ± 10.6 μg/dL and 88.2 ± 21.1 μg/dL, respectively with the difference being statistically insignificant (*p* = 0729). Low zinc levels or zinc deficiency was found in 22 (37.3%) patients from the A subgroup and in 6 (37.5%) patients from the B subgroup (the difference being statistically insignificant; *p* = 0.9824). The results are presented in Table 2 and on Fig. 2.

**Table 2:** Mean serum zinc concentrations in the RAS study group divided into RAS subtypes and in controls

	Zn [μg/dL] Mean ± SD	p	95% CI	Power of the sample
RAS	84.2 ± 13.5	*p* = 0.7154	81.1–87.3	0.054
Controls	83.9 ± 10.0		81.5–86.2	
*RAS subgroups*				
MiRAS	83.5 ± 10.2	*p* = 0.2855	80.8–86.3	0.066
MaRAS	80.0 ± 10.1		74.1–85.9	
HeRAS	103.8 ± 32.3		63.7–143.9	
Mild-type	87.6 ± 18.8	*p* = 0.5032	80.2–95.0	0.089
Moderate-type	82.1 ± 7.5		79.4–84.7	
Severe-type	82.8 ± 12.1		76.1–89.5	
A type	83.1 ± 10.6	*p* = 0.7929	80.4–89.5	0.431
B type	88.2 ± 21.1		76.9–99.4	

Legend:

MiRAS- subgroup with minor aphthous stomatitis

MaRAS- subgroup with major aphthous stomatitis

HeRAS- subgroup with herpetiform aphthous stomatitis

Mild-type- subgroup with mild course of RAS, defined as having episodes less frequently than 3-month intervals

Moderate-type- subgroup with moderate course of RAS, defined as having episodes with 1–3 month intervals

Severe-type- subgroup with severe course of RAS, defined as with lesions almost constantly present

A type- subgroup with 1–3 lesions per episode

B type- subgroup with more than 3 lesions per episode

## Discussion

Aphthous ulcers form in response to an enhanced immunologic reaction against the oral mucosa [3, 25]. An abnormally initiated cascade of cytokines followed by the activation of certain immune processes leads to local tissue damage and inflammation [3, 4, 25]. The disruption of the immune system in RAS predisposed subjects occurs in response to some kind of undefined factors. This may include exposure to viral and bacterial antigens or to some food ingredients, stress or local trauma. The modifying role of certain microelement deficiencies in RAS development has been the subject of several studies, the results however have been rather conflicting. The deficiency in hematins (iron, folic acid, vitamin B12) in RAS subjects was revealed by Khan et al. [26], Lopez-Jornet et al. [27], Natah et al. [1], Scully and Porter [25], and Volkov et al. [28]. However in several other studies the supplementation of deficient microelements modified the course of the disease in only a small percentage of patients (Porter et al. [29]; Nolan et al. [30]; Lalla et al. [31]; Haisraeli-Shalish et al. [32]).

In the present study we have attempted to evaluate the potential role of zinc deficiency as an RAS course modifier. As mentioned in the introduction, zinc deficiency arises with a change in cytokine production [9]. Moreover, low zinc serum levels correlate with a reduced production of Th1-type cytokines [9], while the Th1-type immunologic response is involved in the etiopathogenesis of RAS [3, 25].

The results of our research did not show significant differences in serum zinc levels between RAS patients and generally healthy control subjects. In both groups the mean serum zinc concentrations were within the normal range. The incidence of “deficiency” and “low zinc” patients was similar in both: study and control groups (37.3% vs. 31.9% *p* = 0.4669), with slightly lower zinc concentrations in RAS group compared to the control group. In the Orbak et al. study (2003) low zinc levels were detected in 17 of the 40 examined subjects with RAS (42.5%). However, it should be noted that the authors considered a low zinc concentration as the level below 95 μg/dL, while the norm we suggest ranges from 70 to 100 μg/dL, with a concentration below 70 μg/dL defined as Zn deficiency and levels between 70 and 80 μg/dL defined as low Zn concentrations. In the Orbak study the administration of zinc sulphate in a dose of 220 mg once daily per month resulted in a statistically significant reduction in the frequency of disease recurrence when compared to the subgroup treated with placebo [23]. A reduction in the disease episode frequency was also observed by Endre (1991) who described a 15-years old patient with RAS. His condition improved after a 3-month therapy with zinc sulfate administered orally three times daily in a dose of 50 mg [33]. Some beneficial effects of zinc therapy on the course of RAS were described by Merchant et al. (1977) and by Sharquie et al. (2008) [34, 35]. However, the Wray study (1982) did not confirm any advantages of zinc administration in RAS patients, who also underwent 3-month zinc supplementation [36].

In agreement with our research, Khademi and Shaikhiany (2006), who examined 44 RAS subjects and 44 healthy controls, observed higher serum zinc levels in the control group, but generally in both examined groups the mean zinc levels remained within normal values [19]. No differences in Zn serum levels between RAS and control groups were reported by Arora et al. [12]. Meanwhile, Erel et al. (2003) observed higher serum zinc levels in a study group of 30 patients with Behçet’s disease- a condition characterized by the presence of oral and genital aphthae accompanied by various systemic symptoms- than in 20 healthy controls, but no differences were detected between the patients in the active phase of the disease and in remission [7]. In contrast, significantly lower serum Zn levels were observed in RAS patients compared to healthy controls in the Ozturk et al. study [10].

Experimental studies on rats also revealed that a zinc-deficient diet may affect the oral mucosa and periodontal status. Animals on a zinc-free diet showed a higher incidence of oral ulcers than the control group [37]. Kim et al., who investigated the effects of zinc in a phorbol-12-myristate-13-acetate (PMA)-treated inflammatory model on human gingival fibroblast cells (hGFs), demonstrated that zinc supplement treatment reduced the production of reactive oxygen species (ROS) and decreased the cyclooxygenease-2 (COX-2) expression and prostaglandin E2 (PGE2) release. It was concluded that the zinc treatment resulted in the mitigation of oral inflammation [38].

## Conclusions

The results of several studies indicate that zinc may be at least partially involved in the initiation of RAS or may influence its course and severity. In our research none significant differences were found between the study group and the controls or between the RAS sub-groups. Serum zinc levels did not vary between the patients with more severe types of aphthae (MaRAS) and with less severe forms (MiRAS, HeRAS); it did not influence the mode of recurrences nor the number of lesions per one flare-up.

As described above, the MiRAS patients constituted the core of our study population, while MaRAS and HeRAS were found in 18.7 and 6.7% subjects, respectively. It remains in accordance with the frequency of these types of aphthae in a general population. However, considering the sample size of our study, it makes the MaRAS and HeRAS subgroups not very representative. We are aware of several limitations of this study. The number of patients we managed to examine resulted from the limited study period; also the heterogeneity of our study group, especially in terms of their gender and age was quite high, which could have influence our results. For those reasons the power of the study sample size, as presented in the Table 2, was relatively low. As the risk of false negative results is high in low-powered studies, for more conclusive results a longitudinal research on a larger patients’ sample would be required.

The etiopathogenesis of RAS seems to be due to many genetic, immunologic and environmental factors. Research into zinc supplementation in the treatment of RAS patients has shown some beneficial effects of oral Zn administration. Therefore zinc therapy could be considered as a supportive treatment for RAS. However, in view of the results of our study, further investigations are required to determine if zinc supplementation should be regularly used in the treatment of RAS.

## Abbreviations

CD4, 8, 25: Cluster of differentiation 4, 8, 25
F AAS: Flame atomic absorption spectroscopy
HeRAS: Herpetiform recurrent aphthous stomatitis
IL-1, 2, 6: Interleukin 1, 2, 6
MaRAS: Major recurrent aphthpus stomatitis
MiRAS: Minor recurrent aphthous stomatitis
NK: Natural killer cells
RAS: Recurrent aphthous stomatitis
TCR: T cell receptor
Th1: T1 helper lymphocytes
TNF-α: Tumour necrosis factor alpha
